# Alterations in Sensorimotor and Mesiotemporal Cortices and Diffuse White Matter Changes in Primary Progressive Multiple Sclerosis Detected by Adiabatic Relaxometry

**DOI:** 10.3389/fnins.2021.711067

**Published:** 2021-09-14

**Authors:** Pavel Filip, Michal Dufek, Silvia Mangia, Shalom Michaeli, Martin Bareš, Daniel Schwarz, Ivan Rektor, Lubomír Vojtíšek

**Affiliations:** ^1^Department of Neurology, First Faculty of Medicine and General University Hospital, Charles University, Prague, Czechia; ^2^Center for Magnetic Resonance Research, University of Minnesota, Minneapolis, MN, United States; ^3^First Department of Neurology, Faculty of Medicine, University Hospital of St. Anne, Masaryk University, Brno, Czechia; ^4^Department of Neurology, School of Medicine, University of Minnesota, Minneapolis, MN, United States; ^5^Faculty of Medicine, Institute of Biostatistics and Analyses, Masaryk University, Brno, Czechia; ^6^Institute of Biostatistics and Analyses, Ltd., Masaryk University Spin-Off, Brno, Czechia; ^7^Central European Institute of Technology, Masaryk University, Neuroscience Centre, Brno, Czechia

**Keywords:** primary progressive multiple sclerosis, T1 mapping, T2 mapping, diffusion weighted imaging, DWI, adiabatic T1ρ mapping, adiabatic T2ρ mapping

## Abstract

**Background:** The research of primary progressive multiple sclerosis (PPMS) has not been able to capitalize on recent progresses in advanced magnetic resonance imaging (MRI) protocols.

**Objective:** The presented cross-sectional study evaluated the utility of four different MRI relaxation metrics and diffusion-weighted imaging in PPMS.

**Methods:** Conventional free precession T1 and T2, and rotating frame adiabatic T1ρ and T2ρ in combination with diffusion-weighted parameters were acquired in 13 PPMS patients and 13 age- and sex-matched controls.

**Results:** T1ρ, a marker of crucial relevance for PPMS due to its sensitivity to neuronal loss, revealed large-scale changes in mesiotemporal structures, the sensorimotor cortex, and the cingulate, in combination with diffuse alterations in the white matter and cerebellum. T2ρ, particularly sensitive to local tissue background gradients and thus an indicator of iron accumulation, concurred with similar topography of damage, but of lower extent. Moreover, these adiabatic protocols outperformed both conventional T1 and T2 maps and diffusion tensor/kurtosis approaches, methods previously used in the MRI research of PPMS.

**Conclusion:** This study introduces adiabatic T1ρ and T2ρ as elegant markers confirming large-scale cortical gray matter, cerebellar, and white matter alterations in PPMS invisible to other *in vivo* biomarkers.

## Introduction

The recent years have seen a rapid evolution of advanced magnetic resonance imaging (MRI) techniques in multiple sclerosis (MS) into viable surrogate biomarkers for various pathological processes associated with the disease, be it demyelination, inflammation, or neurodegeneration (Petracca et al., 2018; Cortese et al., 2019). However, the research field seems to be dominated by studies focusing on relapsing–remitting MS (RRMS), vastly overshadowing the primary progressive variant of MS (PPMS). True, a large body of epidemiologic (Simpson et al., 2015), imaging (Rocca et al., 2012), and pathological studies (Lassmann, 2018) position PPMS into the opposite end of the same disease spectrum, but the differences in the dominant clinical phenotypes, clinical course (Antel et al., 2012), and ultimately therapeutic options (Ontaneda et al., 2017) are far from subtle. Also the mechanisms responsible for the development of new focal lesions, a prominent sign in RRMS patients, might differ from the more insidious pathological processes involved in PPMS (Antel et al., 2012). Although lesions are not infrequent in PPMS, the diffuse pathology of both the white and grey matter (WM and GM, respectively) with neurodegeneration is more prominent (Ontaneda et al., 2017). The importance of GM pathology in PPMS, in both subcortical and cortical areas, has been repeatedly emphasized. The cortex suffers from demyelination, microglial activation, and neuronal death but is devoid of perivascular lymphocytic cuffs seen in the WM (Peterson et al., 2001). Indeed, cortical atrophy is prevalent in MS (Fisher et al., 2008), and deep grey structures are not left unaffected in PPMS patients (Anderson et al., 2010; Mesaros et al., 2011). Demyelinated axons, lacking structural, and trophic support of myelin, seem to be more susceptible to chronic injury by inflammatory mediators, reactive oxygen species, and iron compounds, with trans-synaptic degeneration due to distal axonal transection (Trapp et al., 1998).

All the hypothesized mechanisms of neuronal damage, the clinical severity, and tangible progression of the disease are in stark contrast with the paucity of MRI-detected activity in conventional clinical scans. These shortcomings call for the development of more advanced MRI protocols able to distinguish specific pathophysiological processes in PPMS patients. Magnetization transfer ratio (MTR) imaging has demonstrated sensitivity to “occult” WM damage in PPMS not visible to routine T1-weighted (T1w) and T2-weighted (T2w) MRI scans (Leary et al., 1999) and also to GM alterations correlating with clinical disability (Dehmeshki et al., 2003). Furthermore, MTR may be a feasible marker of disease progression in PPMS, as lower baseline normal-appearing WM (NAWM) values have been reported to predict more adverse course of the disease (Khaleeli et al., 2008; Lommers et al., 2019). Also diffusion-weighted imaging (DWI) has been utilized in PPMS, showing differences between PPMS and healthy controls (HCs) in various subcortical structures (Ceccarelli et al., 2009) and diffusely abnormal WM (Vrenken and Geurts, 2007), worsening over time (Rovaris et al., 2005). Also functional MRI studies pointed to non-negligible affection in various domains (Rocca et al., 2010). Despite these advances, the complexity of these methods prevented further spread into the clinical practice; and only a limited number of clinical trials utilized these MRI protocols as endpoints, achieving positive but not very convincing results (Enzinger et al., 2015).

Facing the convoluted situation in PPMS MRI, we have decided to capitalize on the technical developments in adiabatic rotating frame MRI relaxation protocols recently validated as methods receptive to both WM and GM damage in RRMS patients (Filip et al., 2020). The sensitivity of adiabatic T1ρ (Michaeli et al., 2006) and T2ρ (Michaeli et al., 2004) to slow motional regimens detects a different water dynamics range, invisible to conventional protocols. To provide a more complete picture of relaxation metrics abilities in PPMS, we have added both conventional free precession T1 and T2 relaxation mapping protocols due to substantial sensitivity of these techniques in RRMS patients (Bonnier et al., 2017, 2018). The relationships of tissue microstructure and biochemistry with T1 and T2 relaxation time constants, which are particularly sensitive to dipolar fluctuations near the Larmor frequency in the MHz range, and T1ρ and T2ρ, which provide information in the kHz range, should in theory allow for more elaborate identification of eventual pathology.

The primary objective of the presented cross-sectional study was to compare the utility of the above-listed relaxation metrics in PPMS in both GM and WM structures. To this end, high-resolution T1w and T2w scans were utilized for GM/WM segmentation and construction of cortical maps; and separate DWI scans were acquired to enable the reconstruction of relevant WM tracts further utilized as regions of interest (ROIs) for relaxometry analysis. Moreover, NAWM analysis utilizing relaxograms was performed to fully appreciate finer differences detectable by individual relaxation protocols in PPMS patients. The secondary, complementary objective of this study was to evaluate the sensitivity of relaxation protocols against DWI metrics – repeatedly hypothesised as plausible candidates for PPMS monitoring.

## Materials and Methods

### Subjects

Thirteen PPMS patients and thirteen age- and sex-matched HCs were enrolled into this study. The diagnosis of PPMS was based on the latest MAGNIMS criteria (Filippi et al., 2016). Relevant basic neurologic data [Expanded Disability Status Scale (EDSS)], including disease history, were recorded, together with demographic data. The exclusion criteria were presence of MRI contraindications, significant vascular or space-occupying lesions in the MRI scans, and comorbid neurological disorder other than PPMS. Every participant provided a written informed consent in accordance with the Declaration of Helsinki. The study protocol was approved by the ethics committee of the University Hospital of St. Anne.

### Imaging Protocol and Data Analysis

For the full imaging protocol, data analysis, and statistical approach, see the Supplementary Material.

**FIGURE 1:**
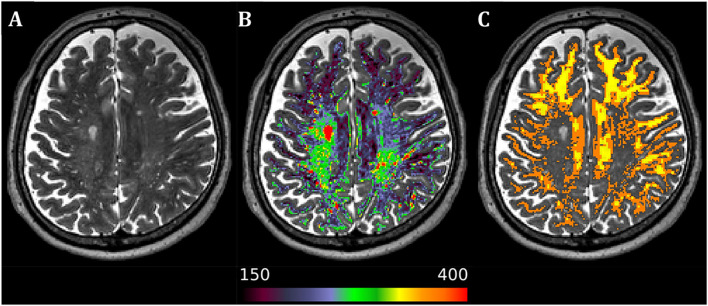
Axial image from a representative primary progressive multiple sclerosis subject. **(A)** Native T2-weighted image. **(B)** Superimposed white matter mask of the T2-weighted image with separate color palette (T2-weighted intensity range 150–400), allowing for easy detection of diffusely abnormal white matter and lesions. **(C)** Resulting normal-appearing white matter mask after manual visual validation and thresholding in 1-mm isotropic resolution of the original T2-weighted scan (orange) and 1.5-mm isotropic resolution of the diffusion-weighted image (yellow) after further inclusion probability thresholding at the level of 0.9 to avoid partial volume effects in lower resolution scans. Laterality conventions where the right side of the figure corresponds to the right side of the brain are used.

Briefly, MRI acquisition was performed in a 3-Tesla Siemens (Erlangen, Germany) Prisma system. The imaging protocol consisted of T1w and T2w high-resolution scans; conventional free precession T1, T2, adiabatic T1ρ, and adiabatic T2ρ maps; and DWI scans. The processing pipeline for structural T1w and T2w images and DWI was based on the Human Connectome Project (HCP) minimal preprocessing pipeline with minor modifications. Processed DWI data were used to calculate the standard diffusion tensor imaging (DTI) parameters (fractional anisotropy (FA), axial diffusivity (AD), radial diffusivity (RD), and mean diffusivity (MD) maps) and mean kurtosis (MK) and to perform probabilistic tractography to reconstruct three main motor function-related tracts – cerebello-thalamo-cortical, cortico-spinal, and cortico-striatal – separately for the left and right sides. Relaxation time constants for T1ρ, T2ρ, and T2 maps were calculated with custom routines utilizing two-parameter non-linear fitting. T1 maps were available as the direct output of the utilized magnetization-prepared 2 rapid gradient-echo (MP2RAGE) sequence for T1w acquisition. NAWM masks were created utilizing a hybrid semiautomatic approach where a T2w intensity threshold was individually selected for each PPMS patient from the FreeSurfer-derived WM ROI in prescan-normalized T2w image (see Figure 1).

The group analysis was performed using separate approaches for GM and WM. In the WM analysis, relevant masks (six tractography-derived masks, FreeSurfer-based whole WM, NAWM mask created as described above) were coregistered to the scans with lower resolution (i.e., T2, T1ρ, T2ρ, FA, AD, RD, and MK) and thresholded to include only voxels with at least 0.9 probability of inclusion in the relevant ROI to limit partial volume effects. Furthermore, we constructed relaxograms (histograms of relaxation time constants) for whole WM in both PPMS and HC and for NAWM in PPMS. For GM analysis of all relevant parameters of interest (T2, T1ρ, T2ρ, FA, MD, and MK), the cortical GM voxels in native space were mapped to cortical surfaces of each subject and resampled to the standard HCP greyordinate - standard terminology space. The Montreal Neurological Institute (MNI)-warped subcortical GM volume images were then combined with cortical surface maps to create CIFTI files for further analysis. While the cross-subject alignment in deep cerebral regions is usually of reasonable precision, this approach benefits from crucial improvement of cortical area correspondence in inter-subject analyses compared with inconsistency-prone MNI coregistration of the cerebral cortex due to high inter-individual variability in cortical folding patterns.

### Statistical Analyses

Two one-sided *t*-test (TOST) procedure was utilized to evaluate equivalence of age between PPMS patients and HC, with 5-year and 33% difference considered significant and the significance level α of the test set at 0.05. Chi-square test was utilized to evaluate absence of significant differences in sex between HC and PPMS groups.

General linear models (GLMs) were used to compare PPMS and HC. Separate GLMs were constructed for the primary objective (T1, T2, T1ρ, and T2ρ maps) and the secondary objective (DWI parameters). For GM analysis, voxel/vertex-wise approach with CIFTI files was utilized; and for WM analysis, median values of relevant ROIs (NAWM, whole WM in a separate model, and six tracks in another separate model) were considered. Median was chosen as the measure of central tendency due to significant departures from normality in multiple metrics. Furthermore, two more GLMs for the analysis of kurtosis in NAWM and whole WM separately for relaxation and for DWI metrics were created. All the GLMs (six in total) included sex and age as covariates of non-interest. And last, we performed a complementary analysis searching for any correlations between EDSS and relevant MRI metrics.

Permutation-based non-parametric analysis as implemented in the Permutation Analysis of Linear Models package (Winkler et al., 2014) was utilized with non-parametric combination (NPC) approach across the individual modalities to perform joint inference (Winkler et al., 2016). For CIFTI files (cortical and deep GM analysis), a type I error of 0.05 was implemented after family-wise error (FWE) voxel/vertex-wise correction, minimal cluster size of 25 voxels (subcortical) and 100 mm^2^ (cortical). For ROI-based WM analysis, we considered the results statistically significant at the predetermined level of *p* < 0.05 with false discovery rate (FDR) correction over modalities and contrasts in each GLM.

## Results

Two one-sided *t*-test and Chi-square test confirmed the equivalence of age and sex distribution, respectively, in PPMS and HC. Demographic information and basic clinical data are provided in Table 1.

**TABLE 1:** Demographics and neurologic data of patients with primary progressive multiple sclerosis and healthy controls.

	PPMS (*n* = 13)	Healthy controls (*n* = 13)
Sex (M/F)	6/7	7/6
Age (years)	60 (40–66)	58 (40–69)
**Neurologic data**		
Age at the onset	46 (35–60)	–
Disease duration	11 (1–30)	–
EDSS	5.5 (3.5–7.5)	–

*The values are stated in the format median (range).*

*PPMS, primary progressive multiple sclerosis; F, female; M, male; EDSS, Expanded Disability Status Scale.*

**FIGURE 2:**
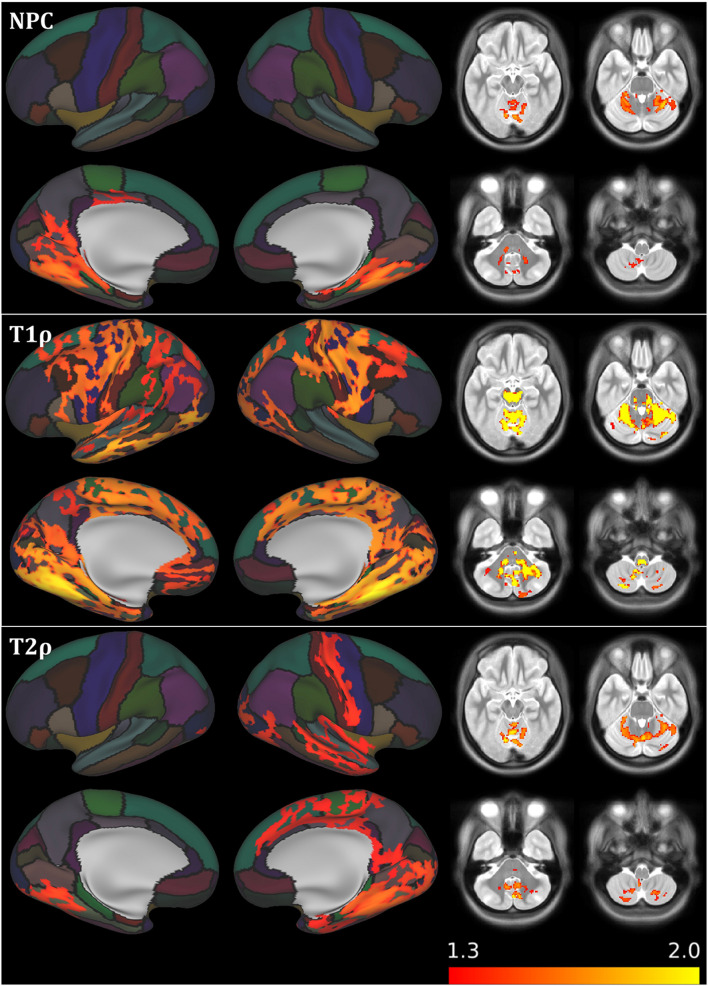
Results of voxel/vertex-wise comparison between primary progressive multiple sclerosis patients and healthy controls for relaxation metrics, including the non-parametric combination joint inference across modalities. Results overlaid over the average FreeSurfer cortical parcellation. Only the results of non-parametric combination (NPC), T1ρ, and T2ρ analysis are presented (T1 map analysis failed to provide significant results, T2 map analysis results not depicted, as only one cortical cluster of limited extent was detected). See Table 2 for further information. Clusters are significant at *p* < 0.05 family-wise error voxel/vertex-wise corrected, cluster threshold of 25 contiguous voxels (subcortical), and 100 mm^2^ (cortical clusters). Results are presented as −log(*p*), with the color range corresponding to *p* < 0.05 and *p* < 0.01 for 1.3 and 2.0, respectively. Laterality conventions where the right side of the figure corresponds to the right side of the brain are used.

The NPC analysis of relaxometry CIFTI data revealed substantial differences between PPMS and HC in the cerebellum and bilateral mesiotemporal cortex (see Table 2 and Figure 2). These alterations were driven by increased relaxation times predominantly in T1ρ (diffuse changes in the whole cerebellum and brainstem and in the primary sensorimotor, premotor, cingulate, and mesiotemporal cortical structures) and T2ρ (the posterior cerebellar lobe, right sensorimotor cortex, and bilateral mesiotemporal cortices). T1 failed to detect any inter-group differences. T2 found only a smaller cluster in the area of the left fusiform gyrus (not depicted in Figure 2). DWI CIFTI analysis was far less fruitful, as only MK was able to detect changes in the right amygdala, hippocampus, and brainstem (see Table 2, not depicted in Figure 2).

**TABLE 2:** Anatomical localization of 3D volume and 2D surface clusters with median (10th–90th percentile) MRI metric values over each cluster.

	Cl. no.	Structure	Median (10th–90th percentile)		Volume (voxels)	−log *p* (FWE)
			
			PPMS	HC		
Relaxometry	**NPC**					
	1	R lob VI, L lob VI	–	–	1,952	1.88
	2	L lob IX, L lob VIIb	–	–	248	1.46
	3	Vermis VIIIa, R lob VIIb	–	–	107	1.47
	**T1ρ map (ms)**					
	1	R lob VI, L lob VI	160 (139–181)	154 (134–172)	7,146	3.11
	**T2ρ map (ms)**					
	1	L lob VI, R lob VI	83 (79–91)	80 (76–86)	3,321	2.20
	2	Brainstem	79 (69–96)	75 (60–87)	60	1.44
DWI	**Mean kurtosis**					
	1	R amygdala	0.65 (0.61–0.71)	0.69 (0.64–0.76)	476	1.62
	2	R hippocampus	0.67 (0.61–0.75)	0.71 (0.65–0.81)	188	1.47
	3	Brainstem	0.86 (0.76–1.05)	0.9 (0.8–1.1)	98	1.51

	**Cl. no.**	**Structure (gyrus)**	**Median (10th–90th percentile)**		**Surface (mm^2^)**	**−log *p* (FWE)**
			
			**PPMS**	**HC**		

Relaxometry	**NPC**					
	1	L fusiform, L lingual, L parahipp	–	–	3,017	1.58
	2	R fusiform, R parahipp	–	–	1,683	1.63
	3	L posterior cingulate	–	–	407	1.34
	**T1ρ map (ms)**					
	1	L precentral, L superior frontal, L postcentral, L fusiform, L cingul	176 (158–207)	168 (154–191)	22,695	1.91
	2	R precentral, R superior frontal, R postcentral, R fusiform, R cingul	174 (157–204)	166 (152–189)	17,717	1.97
	**T2ρ map (ms)**					
	1	R precentral, R postcentral	87 (75–110)	81 (73–98)	5,674	1.37
	2	R superior temporal, R middle temporal	85 (75–103)	81 (73–92)	3,891	1.38
	3	R lateral occipital, R lingual	80 (72–95)	76 (70–86)	3,654	1.55
	4	L lateral occipital	79 (72–90)	73 (69–82)	1,097	1.42
	**T2 map (ms)**					
	1	L fusiform	104 (90–144)	95 (87–111)	885	1.36

*Two GLMs (separately for relaxation and DWI metrics) – permutation analysis with NPC joint inference across modalities. Analyses failing to provide significant results (relaxometry: T1 map; DWI: NPC, FA, and MD) are not provided in the table. Clusters are significant at *p* < 0.05 family-wise error voxel/vertex-wise corrected, cluster threshold of 25 contiguous voxels (subcortical), and 100 mm^2^ (cortical clusters). Only structures providing the highest overlap with individual clusters are listed in the table. GLM, general linear model; FWE, family-wise error; NPC, non-parametric combination; L, left; R, right; lob, cerebellar lobule; DWI, diffusion-weighted imaging.*

**FIGURE 3:**
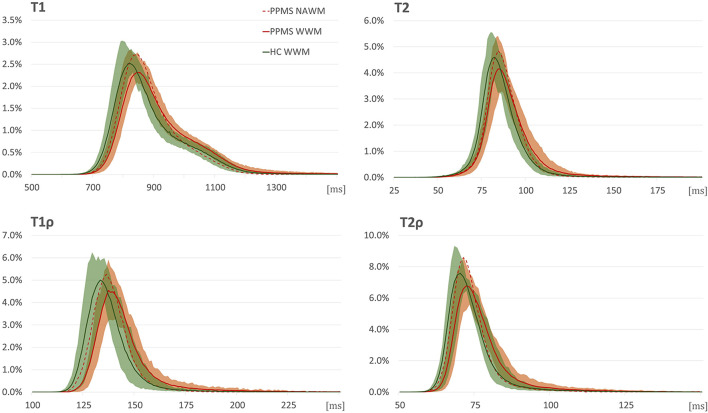
Relaxograms (T1, T2, T1ρ, and T2ρ) in white matter in primary progressive multiple sclerosis (PPMS) patients (red) and healthy controls (HC) (green). Full lines correspond to median values in the whole white matter (WM); shadows to 10th–90th percentile range in the whole WM in the respective group; red dashed line without shadow depicts the median values in normal-appearing white matter in PPMS patients. The *x*-axis provides relaxation time constants in ms; *y*-axis, normalized pixel counts.

White matter analysis detected statistically significantly higher relaxation time constants in PPMS in the whole WM ROI in all the relaxation metrics (see Table 3 and Figure 3), but not in NAWM. Compared with HC, PPMS patients had significantly lower kurtosis in NAWM (mesokurtic in PPMS and leptokurtic in HC), but no inter-group differences in kurtosis were found in whole WM (leptokurtic in both PPMS and HC). On the other hand, DWI metrics failed to show any differences between PPMS and HC, in both the whole WM and NAWM.

**TABLE 3:** Magnetic resonance imaging metrics in whole WM and NAWM.

			Median			−log *p* (FDR)				−log *p* (FDR)	
	ROI	Metrics	(10th–90th percentile)		% Δ	median		Kurtosis		kurtosis	
							
			PPMS	HC		PPMS > HC	HC > PPMS	PPMS	HC	PPMS > HC	HC > PPMS
Relaxometry	Whole WM	NPC	–	–	–	2.25*	0.00	–		0.17	0.17
		T1 (ms)	894 (857–930)	868 (832–908)	3.0	1.58*	0.00	5.11	5.26	0.21	0.00
		T1ρ (ms)	142 (138–148)	136 (132–144)	4.3	1.97*	0.00	2.98	2.91	0.21	0.00
		T2 (ms)	88 (85–93)	85 (81–89)	4.6	1.97*	0.00	5.42	6.02	0.00	0.14
		T2ρ (ms)	76 (73–79)	72 (71–77)	4.7	1.97*	0.00	3.65	4.21	0.00	0.24
	NAWM	NPC	–	–	–	0.19	0.00	–	–	0.00	3.10*
		T1 (ms)	873 (837–897)	868 (832–908)	0.6	0.19	0.00	2.87	5.26	0.00	2.97*
		T1ρ (ms)	138 (134–143)	136 (132–144)	1.2	0.25	0.00	2.08	2.91	0.00	2.97*
		T2 (ms)	87 (83–91)	85 (81–89)	2.8	0.65	0.00	3.19	6.02	0.00	2.97*
		T2ρ (ms)	73 (70–75)	72 (71–77)	1.7	0.13	0.00	2.68	4.21	0.00	2.49*
DWI	Whole WM	NPC	–	–	–	1.18	1.18	–	–	0.50	0.07
		FA	0.44 (0.42–0.48)	0.46 (0.44–0.49)	−5.9	0.00	1.25	1.84	1.83	0.14	0.00
		AD × 10^–3^ (mm^2^ s^–1^)	1.06 (1.01–1.08)	1.03 (0.99–1.08)	2.7	0.81	0.00	2.11	1.97	0.17	0.00
		RD × 10^–3^ (mm^2^ s^–1^)	0.52 (0.47–0.54)	0.49 (0.45–0.51)	7.4	1.25	0.00	2.96	2.65	0.01	0.17
		MK	0.88 (0.84–0.93)	0.91 (0.87–0.96)	−3.1	0.00	1.25	2.40	2.08	0.43	0.00
	NAWM	NPC	–	–	–	0.01	0.46	–	–	0.12	0.34
		FA	0.46 (0.45–0.48)	0.46 (0.44–0.49)	−1.0	0.01	0.06	1.83	1.83	0.24	0.01
		AD × 10^–3^ (mm^2^ s^–1^)	1.02 (0.98–1.05)	1.03 (0.99–1.08)	−0.6	0.00	0.44	1.96	1.97	0.01	0.28
		RD × 10^–3^ (mm^2^ s^–1^)	0.50 (0.46–0.50)	0.49 (0.45–0.51)	2.3	0.06	0.04	1.90	2.65	0.01	0.27
		MK	0.91 (0.88–0.96)	0.91 (0.87–0.96)	−0.1	0.04	0.06	2.36	2.08	0.20	0.01

*Four GLMs (separately for relaxation/DWI metrics and for medians/kurtoses) – permutation analysis with NPC joint inference across modalities. Median (10th–90th percentile) values over each ROI, with percentual differences between PPMS and HC, and non-excess kurtosis are provided, with FDR correction across modalities and contrasts in each GLM; significance level α at 0.05. Statistically significant results are written in bold and marked with an asterisk. Note that the analysis compared NAWM in PPMS patients with whole WM in HC; i.e., the “NAWM” values in HC correspond to respective whole WM values.*

*WM, white matter; NAWM, normal-appearing white matter; GLM, general linear model; PPMS, primary progressive multiple sclerosis; HC, healthy controls; FDR, false discovery rate; DWI, diffusion-weighted imaging; NPC, non-parametric combination; FA, fractional anisotropy; AD, axial diffusivity; RD, radial diffusivity; MK, mean kurtosis.*

In the analysed motor tracts (see Table 4; for complete analyses, see Supplementary Table 1), relaxation metrics were again able to detect significant differences between PPMS and HC, with clear dominance of T1ρ and T2ρ (both significant for all the six considered tracts). Significant inter-group differences in T1 relaxation time constants were found only in the left side tracts. For T2 maps, the inter-group differences reached the predetermined significance threshold only for the left cortico-spinal, left cortico-striatal, and right cortico-thalamo-cerebellar tracts. In the other tractography-derived ROIs, the inter-group comparison of T2 relaxation time constants fell short of surviving the multiple comparison correction. The analysis of DWI metrics did not yield any statistically significant results.

**TABLE 4:** Differences between PPMS and HC in the medians of individual relaxation and DWI metrics over predetermined track masks (cortico-spinal, cortico-striatal, and cortico-thalamo-cerebellar tract, each separately for the left and right hemispheres).

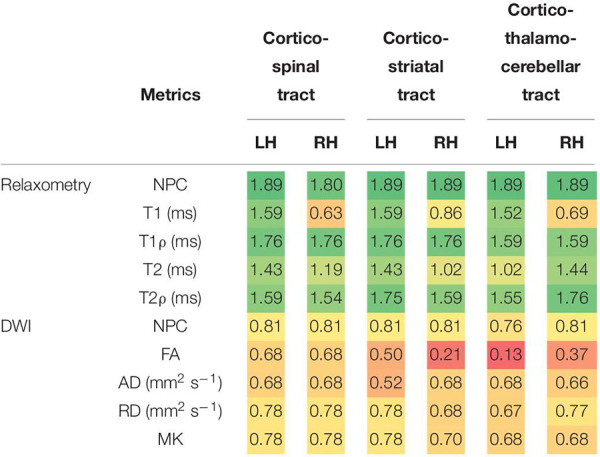

*Two GLMs (separately for relaxation/DWI metrics) – permutation analysis with NPC joint inference across modalities. The table provides −log(*p*) values of relevant tests with FDR correction across modalities and contrasts in each GLM. The statistically significant cells [-log(*p*) > 1.30] are marked in green. The color gradient (red–yellow–green) indicates where each cell falls in the range. Only the dominant direction contrast presented, i.e., PPMS > HC for all relaxometry parameters, NPC, AD, and RD in DWI parameters; and HC > PPMS for FA and MK. For full table including medians and ranges, see Supplementary Table 1.*

*GLM, general linear model; PPMS, primary progressive multiple sclerosis; HC, healthy controls; FDR, false discovery rate; NPC, non-parametric combination; DWI, diffusion-weighted imaging; FA, fractional anisotropy; AD, axial diffusivity; RD, radial diffusivity; MK, mean kurtosis.*

The complementary analysis correlating EDSS and individual MRI metrics failed to reveal any statistically significant correlations at the predetermined alpha.

## Discussion

Despite the dramatic advances in MRI protocols in RRMS, the research in the field of PPMS seems to have been limited by the infrequency of the condition. The consequent limited extent of our knowledge about this disease combined with the bitter paucity of therapeutic options (Ontaneda et al., 2017) clearly calls for targeted research specifically focused on PPMS. The presented study is the first one to evaluate the utility of four different MRI relaxation metrics in combination with DWI parameters in PPMS. T1ρ and T2ρ have proven to be exceptionally sensitive to the differences between PPMS and HC – both in cortical areas and in WM. T1ρ revealed large-scale changes in mesiotemporal structures, the sensorimotor cortex, and the cingulate, in combination with substantial alterations in the WM and cerebellum. T2ρ maps concurred, even though detecting differences of lower extent, but still mimicking the finding in the cerebellum, mesiotemporal structures, and right-side sensorimotor and cingulate cortex.

Surprisingly, these adiabatic relaxation protocols outperformed methods more established in the field of PPMS MRI research, be it T1 and T2 mapping or DTI metrics. Only diffusion kurtosis imaging, a recently developed technique expressing the degree of “non-Gaussianity” of water diffusion (Jensen et al., 2005), found similar changes in the brainstem, but also in the hippocampus and amygdala. FA and diffusivity measures (MD implemented for GM; AD and RD utilized for WM) failed to detect any significant differences in relevant ROIs, partly in contrast with previous reports based on higher numbers of subjects (Rovaris et al., 2002, 2005; Hannoun et al., 2012). Considering the high quality of DWI data utilized in this study with state-of-the art advanced processing, it is highly unlikely that the quality of data would cause the lack of significant findings. Indeed, our study found a 5.9% inter-group difference in FA in the whole WM ROI, but the combination of a relatively low number of PPMS patients with the multiple comparison correction not only within the modality and/or contrast at the level of voxel/vertex-wise analysis but also over several modalities and contrasts implemented in our study (Winkler et al., 2016) probably led to FA falling just short of our predetermined significance level.

The conventional T1 and T2 maps underperformed when compared with the adiabatic T1ρ and T2ρ, too. T1 maps have been proposed as a viable indicator of NAWM affection in PPMS (Manfredonia et al., 2007), based on measures of central tendency and/or histogram shape analysis. In our study, MP2RAGE-derived T1 maps have yielded the least convincing outcomes out of the relaxometry analyses. Before condemning this metric, one should consider the method of calculation utilized by the protocol – MP2RAGE estimates T1 relaxation times and fits the relaxation curve based on two measured points only. Even though it provides expectable ranges in healthy brain tissue, the inferences on the precision of this method in pathologically altered conditions might be premature, and further studies implementing T1 maps reconstructed using more relaxation points might be required to truly validate MP2RAGE-derived T1 maps in pathological conditions.

All in all, adiabatic relaxation protocols were clear winners, confirming their prime position among MRI biomarkers for MS previously established in RRMS (Mangia et al., 2014; Filip et al., 2020). However, while relaxation metrics seem to be exquisitely sensitive to tissue alteration, they are notoriously non-specific, affected by a wide range of processes. Ergo, the results require careful interpretation. T1ρ has been previously associated with neuronal cellular density (Michaeli et al., 2009) – a notion truly intriguing when given into the context of the large-scale cortical T1ρ differences between PPMS and HC, since neuronal and axonal loss seems to be the pathological substrate of progressive disability (Tallantyre et al., 2010) and the reduction of cortico-spinal tract axons, not the extent of demyelination, has been reported to correlate with motor disability (Tallantyre et al., 2009, 2010). The topography of T1ρ differences affecting the primary sensorimotor cortex, premotor cortex, cingulate, and mesiotemporal structures point to widespread alterations consistent with the hypotheses on GM damage (Bodini et al., 2016), with possibly dire clinical implications and interference with a large spectrum of functions. On the other hand, T2ρ has been reported to correlate with iron load (Mitsumori et al., 2009), a trace metal implicated in neurodegeneration (Dusek et al., 2013), oxidative injury leading to mitochondrial dysfunctions in both neurons and glia (Mehta et al., 2013), and mechanisms crucial for the proper function of oligodendrocyte progenitors with possible therapeutic implications (Green et al., 2017). And last, but not least, the acquisition requirements for these methods are much lower than those of high-quality DWI sequences, opening a window for the implementation into the clinical practice.

However, several limitations need to be considered in the context of this study, with the first and most obvious one being the cross-sectional character. Since both the clinical course and the underlying pathophysiological processes in PPMS show certain inter-individual variability (Antel et al., 2012), the need for long-term follow-up studies able to properly assess the progression of the disease and sensitivity of individual methods is dire. Second, we used a relatively rough scale to measure clinical disability, as common in the routine clinical practice. The very character of the scale, with substantial emphasis on the ability to walk in the range above 4.0, makes it a problematic measure for correlation analyses, which presume continuous character of input variables. More complex examinations and scales should definitely be considered in further studies for patient subgrouping and/or localization of presumed damage peaks within the brain. The lack of this information in the presented study does not allow us to elaborate on the nature of several findings, e.g., the apparent laterality of mostly T1 and T2 relaxation metrics in track-derived ROIs. Even though these parameters underperformed in group comparison against HC, this apparent “within-subject” sensitivity to lateral differences might be of non-negligible use under various circumstances. There is of course the possibility to use disease duration as a potential covariate in the utilized GLM, but this approach is far less informative about the progression of the disease and hence presumably the damage to the central nervous system than the clinical score, providing dubious inferences highly confounded by the age of the subject. Third, no formal testing of cognitive deterioration has been performed, which could definitely shed light on the nature and eventual clinical relevance of the substantial alterations in mesiotemporal cortex and cingulate. Nonetheless, it is exceedingly difficult to provide inferences about the deterioration of motor function and higher cognitive processes and their relation to MRI metrics based on cross-sectional studies due to substantial inter-individuality in the disease course. Hence, any plausible hypotheses on causal associations between the detected MRI changes and clinical functional measures should stand on longitudinal data as well. Fourth, the differences in spatial resolution and acquisition times in the presented imaging methods may have led to non-negligible bias, which should be accounted for in future studies. And last, while definitely elegant and versatile for statistical analysis of CIFTI files, as of now, PALM does not provide any effect size estimation to allow for the quantification of the influence of independent variables. Due to its high relevance and informational value, further development of various neuroscientific software packages should consider the inclusion of this metric into their portfolio.

## Conclusion

Advanced MRI techniques are a rapidly evolving field, slowly increasing their value as surrogate biomarkers for relevant pathophysiological processes in virtually all the diseases of the central nervous system. Although still requiring further validation in longitudinal studies with standardized descriptions of motor and cognitive performance and comparisons with well-established clinical MRI markers, T1ρ and T2ρ have been confirmed as elegant markers able to differentiate large-scale cortical GM, cerebellar, and WM alterations. Their ability to detect neuronal loss and iron deposition might be of major importance and provide for suitable outcome measures for future clinical trials in PPMS.

## Data Availability Statement

The raw data supporting the conclusions of this article will be made available by the authors, without undue reservation.

## Ethics Statement

The studies involving human participants were reviewed and approved by Ethics committee of the University Hospital of St. Anne Pekařská 53 656 91 Brno. The patients/participants provided their written informed consent to participate in this study.

## Author Contributions

PF was responsible for the study design, analysis, and interpretation of data, and preparation of manuscript. MD was responsible for study design and patient enrollment, and edited the manuscript. SiM, ShM, and MB participated in the study design and editing of the manuscript. DS was responsible for the statistical analyses and editing of the manuscript. IR edited the manuscript and participated on data interpretation. LV was responsible for the study design, data acquisition, and interpretation, and edited the manuscript. All authors contributed to the article and approved the submitted version.

## Conflict of Interest

DS was employed by the Institute of Biostatistics and Analyses, Ltd. The remaining authors declare that the research was conducted in the absence of any commercial or financial relationships that could be construed as a potential conflict of interest.

## Publisher’s Note

All claims expressed in this article are solely those of the authors and do not necessarily represent those of their affiliated organizations, or those of the publisher, the editors and the reviewers. Any product that may be evaluated in this article, or claim that may be made by its manufacturer, is not guaranteed or endorsed by the publisher.

## Abbreviations

MRI: magnetic resonance imaging
PPMS: primary progressive multiple sclerosis
RRMS: relapsing–remitting multiple sclerosis
GM: gray matter
WM: white matter
HC: healthy controls
NPC: non-parametric combination
FA: fractional anisotropy
MD: mean diffusivity
AD: axial diffusivity
RD: radial diffusivity
MK: mean kurtosis
MNI: Montreal Neurological Institute.
