# Supplementary material for: Alterations in Sensorimotor and Mesiotemporal Cortices and Diffuse White Matter Changes in Primary Progressive Multiple Sclerosis Detected by Adiabatic Relaxometry
Source: Front Neurosci. 2021 Sep 14;15:711067. doi: 10.3389/fnins.2021.711067 (PMC8476998; doi:10.3389/fnins.2021.711067)
Supplement: Supplementary file 1 [file Data_Sheet_1.pdf]

Imaging protocol

A 3 Tesla Siemens Prisma system at the Central European Institute of Technology was utilized for the acquisition. Magnetization-prepared 2 rapid gradient echoes (MP2RAGE) sequence was acquired in sagittal orientation with 1.0 mm isotropic resolution, repetition time (TR) 5,000 ms, echo time (TE) 2,98 ms, inversion times 700 ms (TI1) and 2,500 ms (TI2), flip angle 4° and 5°, generalized autocalibrating partial parallel acquisition (GRAPPA) acceleration factor of 3, acquisition time 8:12 (minutes: seconds). The output of the MP2RAGE protocol includes a pair of images with two inversion times utilized by the software package implemented in the scanner to calculate the T1 map and a T1w image. T2w image was acquired using the SPACE sequence in sagittal orientation also with 1.0 mm isotropic resolution, TR 3,200 ms, TE 412 ms, GRAPPA 2, acquisition time 4:18. DWI sequence utilized the following parameters: 1.5 mm isotropic resolution, TR 3,222 ms, TE 89.20 ms, multi-band acceleration factor 4, b-values of 1,500 and 3,000 s/mm^2 in 93 directions, with 7 additional b0 images [Harms et al., 2018]. The DWI acquisitions were performed twice with opposite phase encoding along the antero-posterior axis, acquisition time 11:20 in total. Furthermore, multi-slice-multi-echo T2 mapping sequence was acquired: voxel size 1.0 × 1.0 × 3.5 mm, TR 7,020 ms, GRAPPA 2, with 17 echo times from 10 to 170 ms with ΔTE spacing of 10 ms, acquisition time 9:04. Adiabatic rotating frame relaxometric sequences T1ρ and T2ρ were obtained with the following parameters: voxel size 1.6 × 1.6 × 3.5 mm, TR 2,000 ms, TE 2,82 ms, GRAPPA 3, for T1ρ and T2ρ preparation portion of the sequence adiabatic full passage (AFP) hyperbolic secant (HS1) pulses were used [Garwood and DelaBarre, 2001], adiabaticity factor R = 10, pulse duration = 6 ms, number of pulses = 0, 4, 8, 12, 16 phase cycled according to MLEV-4 [Levitt et al., 1982], bandwidth = 1.3 kHz, peak power $\omega1^{max}/(2\pi)$ = 800 Hz. For adiabatic T1ρ measurements train of HS1 pulses was placed prior to the readout portion of the sequence when magnetization oriented along the +Z was not perturbed while for adiabatic T2ρ relaxation mapping train of AFP HS1 pulses was placed after the adiabatic half passage (AHP) pulse used for coherent excitation, and then returned back to +Z using AHP - reverse pulse according to the previously described protocols [Mangia et al., 2017]. Acquisition times were 7:23 for T1ρ and T2ρ protocols, each. All the sequences covered the entire brain including the cerebellum and brainstem.

Image analysis

In the first step preceding the automatic analysis, “lesion filling” process [Battaglini et al., 2012] was used to mask the WM “black holes” in the T1w scan of PPMS subjects, which would otherwise lead to substantial errors in FreeSurfer-based segmentation. The following processing pipeline for structural T1w and T2w images was based on the human connectome project (HCP) minimal preprocessing pipeline [Glasser et al., 2013] with minor modifications. Namely, brain mask extraction in the PreFreeSurfer step utilized the TI2 output of the MP2RAGE sequence, while further steps were performed in the combined T1w output of the MP2RAGE sequence [Marques et al., 2010]. The high noise in the background in the combined T1w MP2RAGE image would otherwise lead to substantial imprecision of brain mask extraction, while the combined T1w MP2RAGE image provided much higher GM/WM contrast optimal for the subsequent FreeSurfer step. The FreeSurfer step of the HCP pipeline used CUDA (Compute Unified Device Architecture)-enabled version of FreeSurfer 6.0 (http://surfer.nmr.mgh.harvard.edu). The original HCP PostFreeSurfer step was implemented afterwards without any changes. The accuracy of FreeSurfer segmentation was visually inspected by P.F.

NAWM masks were created utilizing a hybrid semiautomatic approach where a T2w intensity threshold was individually selected for each PPMS patient from the FreeSurfer-derived WM ROI in prescan-normalized T2w image (see Fig. 1).

DWI processing also followed the HCP minimal preprocessing pipeline, including the optional gradient non-linearity correction. Afterwards, diffusion tensor model was fitted to generate fractional anisotropy (FA), axial diffusivity (AD), radial diffusivity (RD) and mean diffusivity (MD) maps. To avoid the deviation from the mono-exponential model of intravoxel incoherent motion, DWI scans with the b-value of 3,000 mm/s^2 were not utilized for diffusion tensor fitting. Mean kurtosis (MK) map [Jensen et al., 2005] was calculated utilizing all the acquired gradients. And lastly, probabilistic tractography was run – Bedpostx toolbox [Jenkinson et al., 2012] (3 fibres per voxel, “zeppelin” single-fibre response kernel for deconvolution and Rician noise model, 3,000 burn-in iterations, with gradient non-linearity correction). 3 main motor function related tracts were estimated using fibre tracking in the native space of each subject – cerebello-thalamo-cortical, cortico-spinal and cortico-striatal, all of them separately for the left and the right side (i.e. six tracks were reconstructed in each subject). In all the tracks, the seed was positioned in the precentral gyrus. For the cerebello-thalamo-cortical tract, the waypoints were located in the ipsilateral thalamus and cerebellar grey matter, in the given order, and the termination mask in the cerebellar grey matter. The cortico-spinal and cortico-striatal tract were modelled with waypoints and simultaneously termination masks in the medulla and striatum, respectively. Medulla mask was derived from auxiliary brainstem sub-segmentation [Iglesias et al., 2015], the other listed masks were based on standard FreeSurfer segmentation/parcellation in the native space. In all the track reconstructions, pial surfaces were used as termination masks to avoid spurious tracks “jumping” over gyri surfaces. FreeSurfer-derived midline corpus callosum mask was set as an exclusion masks to avoid artefactual, anatomically incorrect midline crossings of tracks via the corpus callosum. The lower threshold of 100 was used to remove the less robust fibre tracks and the resulting track mask was binarized for the follow-up region of interest (ROI) analysis of relaxation and DWI metrics.

The processing of T1ρ, T2ρ and T2 maps utilized one pipeline: after 3D rigid-body motion correction of all the acquired scans to the first scan of each of these sequences (trilinear interpolation, mutual information as cost function followed by an optimization pass with sinc interpolation as implemented in the FSL 6.0 MCFLIRT), relaxation time constants were calculated utilizing 2-parameter non-linear fitting (custom routines in MATLAB R2016a; MathWorks, Inc., Natick, MA). Afterwards, each map was co-registered to the “HCP space” – mri_robust_register-initialized BB-register algorithm and subsequent FSL 6.0 FLIRT initialized with the relevant matrix as provided by the HCP pipeline from the FreeSurfer space to the “HCP space”.

Visual inspection and evaluation of root-mean-squared voxel displacement for motion-correction in T1ρ, T2ρ and T2 maps reconstruction and DWI set revealed no motion exceeding the extent of two voxels in any of the subjects.

The group analysis was performed using separate approaches for GM and WM. In the WM analysis, relevant masks (6 tractography-derived masks, FreeSurfer-based whole WM, NAWM mask created as described above) were co-registered to the scans with lower resolution (i.e. T2, T1ρ, T2ρ, FA, AD, RD, MK) utilizing inverse matrices to the matrices generated by the coregistration of individual scans to the HCP space (see above). These coregistered masks were then thresholded to include only voxels with at least 0.9 probability of inclusion in the relevant ROI to limit partial volume effects. Furthermore, we constructed relaxograms (histograms of relaxation time constants) for whole WM in both PPMS and HC and for NAWM in PPMS.

For GM analysis, all relevant volumes of interest (T2, T1ρ, T2ρ, FA, MD, MK) were masked to exclude cortical GM and subcortical WM voxels. These “decorticated” images were warped to the MNI space utilizing the HCP-pipeline derived matrices and resampled to 2-mm isotropic resolution. The cortical GM voxels in native space were mapped to cortical surfaces of each

subject and resampled to the standard HCP greyordinate space. The subcortical GM volume images were then connected to the cortical surface maps to create CIFTI files for further analysis. While the cross-subject alignment in deep cerebral regions is usually of reasonable precision, this approach benefits from crucial improvement of cortical area correspondence in inter-subject analyses compared to inconsistency-prone MNI coregistration of cerebral cortex due to high inter-individual variability in cortical folding patterns. Hence, only lower level of spatial smoothing is necessary (4-mm full-width half-maximum Gaussian kernel), thus retaining localized information in higher extent than the generally used larger smoothing kernels [Van Essen et al., 2012].

Contrary to the GM analysis, WM analysis for the second objective distinguished AD and RD due to the hypothesized significance of the parameters for axonal and myelin integrity, respectively. As these AD/RD-based inferences are of rather dubious nature in GM, GM analysis utilized only MD.

Harvard-Oxford subcortical structural atlas and the probabilistic cerebellar atlas [Diedrichsen et al., 2009] were used for the classification of significant clusters in the MNI space and the average FreeSurfer parcellation atlas for significant clusters in cortical maps.

Statistical analyses

Two one-sided t-test (TOST) procedure was utilized to evaluate equivalence of sex and age between PPMS patients and HC, with the significance level α of the test set at 0.05.

General linear models (GLMs) were used to compare PPMS and HC. Separate GLMs were constructed distinguishing between the primary objective (all the relaxation metrics – T1, T2, T1ρ, T2ρ maps) and the secondary objective (DWI parameters), and furthermore, between GM and WM (here distinguishing a separate model for NAWM and whole WM and a separate model for the 6 above stated preselected tracks). Altogether, 6 main GLMs were utilized – relaxometry in GM, relaxometry in WM comparing NAWM and whole WM, relaxometry in WM comparing the 6 preselected tracks, DWI in GM, DWI in WM comparing NAWM and whole WM and DWI in WM comparing the 6 preselected tracks). For GM analysis, voxel/vertex-wise approach with CIFTI files was utilized and for WM analysis, median values of relevant ROIs (NAWM, whole WM in a separate model and 6 tracks in another separate model) were considered. Median was chosen as the measure of central tendency since Anderson-Darling tests revealed significant departures from normality in multiple metrics [Stephens, 1974]. Furthermore, 2 more GLMs for the analysis of kurtosis in NAWM and whole WM separately for relaxation and for DWI metrics was created. All the GLMs (6 in total) included sex and age as covariates of non-interest. And lastly, we performed a complementary analysis searching for any correlations between EDSS and relevant MRI metrics, utilizing six separate GLMs only for the PPMS group (again, a model for voxel/vertex-wise analysis for GM, a model comparing medians over NAWM and whole WM and a model comparing the medians over 6 preselected tracts, each separately for DWI and relaxation metrics).

Permutation-based non-parametric analysis as implemented in the Permutation Analysis of Linear Models package [Winkler et al., 2014] was utilized with non-parametric combination (NPC) approach across the individual modalities (Fisher method as combining function) to perform joint inference [Winkler et al., 2016]. 5,000 permutations were run. CIFTI files employed threshold-free cluster enhancement [Smith and Nichols, 2009] and adjustment over the average area per vertex in the surface maps.

For CIFTI files (cortical and deep GM analysis), a type I error of 0.05 was implemented after family-wise error (FWE) voxel/vertex-wise correction, minimal cluster size of 25 voxels (subcortical) and 100 mm^2 (cortical). For ROI-based WM analysis, we considered the results

statistically significant at the predetermined level of $p < 0.05$ with false discovery rate (FDR) correction [Benjamini and Hochberg, 1995] over modalities and contrasts in each GLM model.

Supplementary tables

Supplementary table 1: MRI metrics in the predetermined track masks; 2 GLMs (separately for relaxation/DWI metrics) – permutation analysis with NPC joint inference across modalities. Median [10th–90th percentile] values over each ROI, with percentual differences between PPMS and HC. FDR correction across modalities and contrasts in each GLM, with the significance level α at 0.05. Statistically significant results written in bold and marked with an asterisk. Abbreviations: GLM – general linear model; PPMS – primary progressive multiple sclerosis; HC – healthy controls; FDR – false discovery rate; NPC – non-parametric combination; DWI – diffusion weighted imaging; FA – fractional anisotropy; AD – axial diffusivity; RD – radial diffusivity; MK – mean kurtosis

	ROI	Metrics	Median [10th-90th percentile]		% Δ	– log p (FDR) median	
			PPMS	HC		PPMS > HC	HC > PPMS
**Relaxometry**	Left cortico-spinal	NPC	–	-	–	**1.89***	0.00
		T1 *[ms]*	918 [894–978]	894 [860–929]	2.7%	**1.59***	0.00
		T1ρ *[ms]*	148 [144–162]	140 [137–151]	5.4%	**1.76***	0.00
		T2 *[ms]*	97 [94–105]	94 [91–100]	4.0%	**1.43***	0.00
		T2ρ *[ms]*	80 [77–87]	77 [75–82]	3.8%	**1.59***	0.00
	Left cortico-striatal	NPC	918 [886–1013]	891 [859–926]	3.0%	**1.89***	0.00
		T1 *[ms]*	148 [144–164]	140 [137–152]	5.4%	**1.59***	0.00
		T1ρ *[ms]*	96 [90–103]	91 [88–98]	4.9%	**1.76***	0.00
		T2 *[ms]*	79 [76–85]	75 [74–80]	4.7%	**1.43***	0.00
		T2ρ *[ms]*	918 [886–1013]	891 [859–926]	3.0%	**1.75***	0.00
	Left cortico-thalamo-cerebellar	NPC	–	-	–	**1.89***	0.00
		T1 *[ms]*	932 [904–960]	909 [874–936]	2.5%	**1.52***	0.00
		T1ρ *[ms]*	148 [144–156]	141 [138–151]	4.5%	**1.59***	0.00
		T2 *[ms]*	97 [93–102]	93 [91–99]	4.1%	1.02	0.00
		T2ρ *[ms]*	80 [77–83]	77 [75–81]	4.1%	**1.55***	0.00
	Right cortico-spinal	NPC	–		–	**1.80***	0.00
		T1 *[ms]*	900 [876–927]	886 [863–924]	1.6%	0.63	0.00
		T1ρ *[ms]*	146 [143–152]	140 [138–149]	4.2%	**1.76***	0.00
		T2 *[ms]*	96 [94–103]	93 [91–100]	2.9%	1.19	0.00
		T2ρ *[ms]*	80 [77–84]	77 [75–82]	3.9%	**1.54***	0.00
	Right cortico-striatal	NPC	–	-	–	**1.89***	0.00
		T1 *[ms]*	892 [869–927]	881 [855–924]	1.2%	0.86	0.00
		T1ρ *[ms]*	146 [143–153]	140 [138–149]	4.3%	**1.76***	0.00
		T2 *[ms]*	95 [91–101]	92 [89–97]	3.1%	1.02	0.00
		T2ρ *[ms]*	79 [76–82]	75 [74–80]	4.6%	**1.59***	0.00
	Right cortico-thalamo-cerebellar	NPC	–	-	–	**1.89***	0.00
		T1 *[ms]*	915 [899–946]	908 [861–954]	0.8%	0.69	0.00
		T1ρ *[ms]*	148 [143–153]	141 [137–150]	4.4%	**1.59***	0.00
		T2 *[ms]*	96 [93–102]	94 [91–97]	2.3%	**1.44***	0.00
		T2ρ *[ms]*	79 [77–84]	77 [75–81]	2.9%	**1.76***	0.00
**DWI**	Left cortico-spinal	NPC	–	-	–	0.81	0.81
		FA	0.46 [0.43–0.48]	0.47 [0.44–0.50]	-2.8%	0.00	0.68
		AD $\times 10^{-3}$ *[$mm^2 s^{-1}$]*	1.01 [0.96–1.08]	0.98 [0.95–1.03]	2.9%	0.68	0.00
		RD $\times 10^{-3}$ *[$mm^2 s^{-1}$]*	0.48 [0.45–0.50]	0.45 [0.43–0.48]	6.0%	0.78	0.00
		MK	0.93 [0.88–0.98]	0.96 [0.93–1.00]	-3.7%	0.00	0.78
	Left cortico-striatal	NPC	–	-	–	0.81	0.81
		FA	0.40 [0.36–0.42]	0.40 [0.38–0.44]	0.3%	0.00	0.68
		AD $\times 10^{-3}$ *[$mm^2 s^{-1}$]*	0.96 [0.91–1.03]	0.94 [0.91–0.98]	2.1%	0.68	0.00
		RD $\times 10^{-3}$ *[$mm^2 s^{-1}$]*	0.51 [0.47–0.54]	0.50 [0.47–0.51]	2.8%	0.78	0.00
		MK	0.89 [0.84–0.95]	0.92 [0.89–0.97]	-3.4%	0.00	0.78
		NPC	–		–	0.81	0.81

	Left cortico-thalamo-cerebellar	FA	0.43 [0.40–0.46]	0.43 [0.41–0.50]	0.5%	0.00	0.50
		AD $\times 10^{-3}$ *[mm²s⁻¹]*	0.99 [0.92–1.03]	0.95 [0.92–1.01]	4.1%	0.52	0.00
		RD $\times 10^{-3}$ *[mm²s⁻¹]*	0.48 [0.45–0.50]	0.46 [0.43–0.49]	5.0%	0.78	0.00
		MK	0.93 [0.90–1.01]	0.98 [0.93–1.01]	-5.2%	0.00	0.78
	Right cortico-spinal	NPC	–	-	–	0.81	0.47
		FA	0.45 [0.44–0.49]	0.47 [0.45–0.49]	-2.5%	0.00	0.21
		AD $\times 10^{-3}$ *[mm²s⁻¹]*	1.02 [0.94–1.06]	0.99 [0.94–1.02]	3.2%	0.68	0.00
		RD $\times 10^{-3}$ *[mm²s⁻¹]*	0.47 [0.45–0.49]	0.46 [0.44–0.48]	1.3%	0.68	0.00
		MK	0.92 [0.89–0.98]	0.95 [0.91–0.99]	-3.2%	0.00	0.68
	Right cortico-striatal	NPC	–		–	0.76	0.51
		FA	0.40 [0.39–0.43]	0.40 [0.39–0.44]	-0.9%	0.00	0.13
		AD $\times 10^{-3}$ *[mm²s⁻¹]*	0.98 [0.90–1.02]	0.94 [0.91–0.98]	3.9%	0.68	0.00
		RD $\times 10^{-3}$ *[mm²s⁻¹]*	0.50 [0.48–0.53]	0.49 [0.47–0.52]	1.8%	0.67	0.00
		MK	0.89 [0.86–0.95]	0.92 [0.88–0.96]	-2.9%	0.00	0.70
	Right cortico-thalamo-cerebellar	NPC	–	-	–	0.81	0.59
		FA	0.42 [0.40–0.46]	0.44 [0.39–0.48]	-4.5%	0.00	0.37
		AD $\times 10^{-3}$ *[mm²s⁻¹]*	0.97 [0.90–1.01]	0.95 [0.93–0.98]	2.9%	0.66	0.00
		RD $\times 10^{-3}$ *[mm²s⁻¹]*	0.48 [0.46–0.50]	0.46 [0.44–0.49]	3.2%	0.77	0.00
		MK	0.94 [0.90–0.99]	0.97 [0.92–1.01]	-2.5%	0.00	0.68

Supplementary references

Battaglini M, Jenkinson M, De Stefano N (2012): Evaluating and reducing the impact of white matter lesions on brain volume measurements. Human brain mapping 33:2062–2071.

Benjamini Y, Hochberg Y (1995): Controlling the False Discovery Rate: A Practical and Powerful Approach to Multiple Testing. Journal of the Royal Statistical Society Series B (Methodological) 57:289–300.

Diedrichsen J, Balsters JH, Flavell J, Cussans E, Ramnani N (2009): A probabilistic MR atlas of the human cerebellum. NeuroImage 46:39–46.

Garwood M, DelaBarre L (2001): The return of the frequency sweep: designing adiabatic pulses for contemporary NMR. Journal of magnetic resonance 153:155–177.

Glasser MF, Sotiropoulos SN, Wilson JA, Coalson TS, Fischl B, Andersson JL, Xu J, Jbabdi S, Webster M, Polimeni JR (2013): The minimal preprocessing pipelines for the Human Connectome Project. Neuroimage 80:105–124.

Harms MP, Somerville LH, Ances BM, Andersson J, Barch DM, Bastiani M, Bookheimer SY, Brown TB, Buckner RL, Burgess GC, Coalson TS, Chappell MA, Dapretto M, Douaud G, Fischl B, Glasser MF, Greve DN, Hodge C, Jamison KW, Jbabdi S, Kandala S, Li X, Mair RW, Mangia S, Marcus D, Mascali D, Moeller S, Nichols TE, Robinson EC, Salat DH, Smith SM, Sotiropoulos SN, Terpstra M, Thomas KM, Tisdall MD, Ugurbil K, van der Kouwe A, Woods RP, Zöllei L, Van Essen DC, Yacoub E (2018): Extending the Human Connectome Project across ages: Imaging protocols for the Lifespan Development and Aging projects. NeuroImage 183:972–984.

Iglesias JE, Van Leemput K, Bhatt P, Casillas C, Dutt S, Schuff N, Truran-Sacrey D, Boxer A, Fischl B, Initiative ADN (2015): Bayesian segmentation of brainstem structures in MRI. Neuroimage 113:184–195.

Jenkinson M, Beckmann CF, Behrens TE, Woolrich MW, Smith SM (2012): Fsl. Neuroimage 62:782–790.

Jensen JH, Helpern JA, Ramani A, Lu H, Kaczynski K (2005): Diffusional kurtosis imaging: The quantification of non-gaussian water diffusion by means of magnetic resonance imaging. Magnetic Resonance in Medicine 53:1432–1440.

Levitt MH, Freeman R, Frenkiel T (1982): Broadband heteronuclear decoupling. Journal of Magnetic Resonance (1969) 47:328–330.

Mangia S, Svatkova A, Mascali D, Nissi MJ, Burton PC, Bednarik P, Auerbach EJ, Giove F, Eberly LE, Howell MJ (2017): Multi-modal Brain MRI in Subjects with PD and iRBD. Frontiers in neuroscience 11.

Marques JP, Kober T, Krueger G, van der Zwaag W, Van de Moortele P-F, Gruetter R (2010): MP2RAGE, a self bias-field corrected sequence for improved segmentation and T1-mapping at high field. NeuroImage 49:1271–1281.

Smith SM, Nichols TE (2009): Threshold-free cluster enhancement: addressing problems of smoothing, threshold dependence and localisation in cluster inference. Neuroimage 44:83–98.

Stephens MA (1974): EDF Statistics for Goodness of Fit and Some Comparisons. Journal of the American Statistical Association 69:730–737.

Van Essen DC, Ugurbil K, Auerbach E, Barch D, Behrens TEJ, Bucholz R, Chang A, Chen L, Corbetta M, Curtiss SW (2012): The Human Connectome Project: a data acquisition perspective. Neuroimage 62:2222–2231.

Winkler AM, Ridgway GR, Webster MA, Smith SM, Nichols TE (2014): Permutation inference for the general linear model. NeuroImage 92:381–397.

Winkler AM, Webster MA, Brooks JC, Tracey I, Smith SM, Nichols TE (2016): Non-parametric combination and related permutation tests for neuroimaging. Human brain mapping 37:1486–1511.
